# Proton GRID and LATTICE treatment planning techniques for clinical liver SFRT treatments

**DOI:** 10.1088/1361-6560/add2cc

**Published:** 2025-05-19

**Authors:** Jufri Setianegara, Ya-Nan Zhu, Mingyao Zhu, Rajeev Badkul, Tianyu Zhao, Harold Li, Fen Wang, David Akhavan, Hao Gao, Yuting Lin

**Affiliations:** 1 Department of Radiation Oncology, University of Pennsylvania, Philadelphia, Pennsylvania, United States of America; 2 Department of Radiation Oncology, University of Kansas Medical Center, Kansas City, Kansas, United States of America; 3 Department of Radiation Oncology, Emory University, Atlanta, Georgia, United States of America; 4 Department of Radiation Oncology, University of South Florida, Tampa, Florida, United States of America

**Keywords:** proton spatially fractionated radiotherapy (pSFRT), proton GRID (pGRID), proton LATTICE (pLATTICE), radiotherapy, liver hepatocellular carcinoma, generalized equivalent uniform dose (gEUD)

## Abstract

*Objective*. This study aims to develop and evaluate various treatment clinical proton spatially-fractionated-radiotherapy (pSFRT) planning techniques namely proton GRID (pGRID) and LATTICE (pLATTICE). *Approach.* pSFRT plans (27 Gy(RBE), single-fraction) were initially developed using phantom geometries and created retrospectively for a liver patient previously treated with photon virtual GRID (vGRID). pGRID plans varied in cylinder diameters (*D*_cyl_ = 0.4–1.4 cm) and center-to-center distances (c-t-c = 1.7–3.4 cm) and were evaluated by peak-to-valley dose ratios (PVDRs), equivalent uniform dose (EUD), and *V*_27Gy_. *D*_cyl_ and c-t-c distances matching the vGRID EUD and *V*_27Gy_ values guided pLATTICE sphere distributions. Various pLATTICE techniques, including different beam numbers, orientations, and sphere arrangements, were investigated. We also explored using collimating brass apertures to enhance the pGRID PVDR. *Main results.* pGRID plans with 3.4 cm c-t-c and 0.4 cm *D*_cyl_ resulted in 2.09% *V*_27Gy_, closely matching vGRID’s 1.50%. The resultant pGRID PVDR was 8.92 compared to vGRID’s 2.7–3.0. PVDRs were affected by spot sizes with reductions of 15.0% with range shifters and 76.0% from 7.5 cm to 27.5 cm depths. The highest PVDR of 4.17 was achieved with two-field pLATTICE plans with favorable beam angles, with a 44.6% reduction with unfavorable beam orientations and up to 24.7% reductions with an increasing number of beams. Non-maximal pLATTICE sphere packing arrangements increases the PVDR with a decrease in *V*_27Gy_ warranting further investigation. pSFRT plans reduced the healthy liver *V*_5Gy_ by 83.6%–90.7% compared to vGRID. Apertures enhanced the PVDR by 170% at the deepest depths but increased the skin D_0.03cc_ from 26.77 Gy to 54.66 Gy. *Significance and conclusion.* We developed pGRID and pLATTICE plans, demonstrating that desired plan metrics was achieved by adjusting the geometrical arrangements of SFRT contours. The relative orientation of these contours with beam entrances was crucial for high-quality SFRT plans. pSFRT plans achieved superior PVDRs and better dose sparing to OARs compared to vGRID plans.

## 1. Introduction

Spatially fractionated radiotherapy (SFRT) treatments are a novel radiation technique designed to increase the therapeutic ratio of radiation therapy by intentionally creating a highly heterogeneous dose distribution within the tumor subvolume (Billena and Khan 2019, Yan *et al* 2020, Prezado 2022, Mali 2024, Prezado *et al* 2024). Historically, SFRT techniques such as GRID were used to minimize skin toxicities often associated with the treatment of deep-seated tumors (Yan *et al* 2020). Although the use of SFRT decreased with the advent of modern megavoltage linear accelerators that offer better skin sparing, these techniques have experienced a resurgence in the palliative treatment of bulky tumors (Mohiuddin *et al* 1990, 1996, 1999). The safety and efficacy of SFRT relative to conventional treatments have been demonstrated in the debulking of large tumors (Zhang *et al* 2020) while controlling normal tissue toxicities (Peñagarícano *et al* 2010) typically associated with delivering therapeutic radiation doses to a large tumor volume. While the exact biological mechanisms underpinning the efficacy of novel SFRT treatments are not definitively known (Zhang *et al* 2020, Prezado *et al* 2024), several viable hypotheses have been presented. These include bystander and abscopal effects (Kanagavelu *et al* 2014, Johnsrud *et al* 2020), tumor microvascular changes (Bouchet *et al* 2015, Kozin 2022, Song *et al* 2023) and most recently, radiation-induced immune-responses (Bazyar *et al* 2021, Jiang *et al* 2021, Bertho *et al* 2023a, 2023b). Preclinical evidence has shown promising results of intratumoral immune cell sparing in SFRT-treated mice (Lukas *et al* 2023), with the upregulation of PD-L1 and increased numbers of CD4+ and CD8+ T cells in animal models (Johnsrud *et al* 2020, Lukas *et al* 2023).

Currently, thousands of patients have received SFRT treatments, with the vast majority performed using photons primarily using the 2D GRID approach and occasionally with 3D LATTICE techniques (Mayr *et al* 2024). Traditionally, SFRT was delivered using a single unopposed field, administering a photon dose of 10–15 Gy with spatial modulation achieved through hexagonal apertures milled within brass attenuating blocks (Zhang *et al* 2024). These 2D techniques are commonly referred to as ‘GRID’ treatments and offer the advantage of relatively simple treatment planning and delivery. More sophisticated SFRT planning techniques utilizing multi-leaf collimators (MLCs) have since been developed for GRID deliveries (Grams *et al* 2023). LATTICE radiotherapy represents a more advanced form of SFRT treatment planning and delivery, creating spatial modulation in 3D using intensity-modulated radiation therapy (IMRT) techniques (Wu *et al* 2020, Duriseti *et al* 2021, Grams *et al* 2022, Kavanaugh *et al* 2022). There is increasing interest in developing and investigating the clinical efficacy of proton SFRT (pSFRT) treatments using proton GRID (pGRID) and proton LATTICE (pLATTICE) techniques (Henry *et al* 2017, Gao *et al* 2018, Mohiuddin *et al* 2020, Grams *et al* 2022, Yang *et al* 2022, Zhang *et al* 2023, Mossahebi *et al* 2024). pSFRT has the potential to produce better abscopal immunologic effects by creating higher peak-to-valley dose ratios (PVDR) within the tumor and more efficient dose sparing for intratumoral and adjacent organs at risk (OAR). Additionally, proton pencil beam scanning units with smaller spot sizes allow for increased customizability of proton SFRT plans in an efficient manner.

Despite its promise, pSFRT treatments are not as well-studied due to the technical complexities associated with creating clinically deliverable plans with comparable SFRT plan metrics (Mayr *et al* 2024). The purpose of this work is to: (i) comprehensively develop treatment planning methodologies, specifically pGRID and pLATTICE, to enable clinical proton SFRT treatments on a compact proton machine (IBA Proteus®ONE); (ii) systematically investigate the differences in plan qualities and SFRT metrics across various SFRT planning techniques; and (iii) cross-compare these proton SFRT plans with their photon SFRT counterparts in a clinical patient case.

## 2. Methods and materials

### 2.1. Treatment planning system and beam model

All treatment planning in this study was conducted using the RayStation v2023B treatment planning system (RaySearch Laboratories, Stockholm, Sweden), incorporating a proton beam model that represents the IBA Proteus®ONE unit at the University of Kansas Proton Center. The IBA Proteus®ONE is a compact, single-gantry proton unit equipped with a superconducting synchrocyclotron (S2C2) that delivers proton beams with a nominal energy of 230 MeV. The synchrocyclotron operates at a pulse repetition rate of 1 kHz, with a pulse duration of 10 *μ*s and a maximum charge of 4.5 pC per proton pulse at the isocenter (Pearson *et al* 2013). A variable energy degrader located at the cyclotron’s exit enables the reduction of proton energies from 230 MeV to 70 MeV. Central to the IBA Proteus®ONE’s design is the integration of the energy selection system within the gantry, which includes divergence-limiting slits to restrict the beam emittance to the central region of the beam spot.

The compact gantry of the IBA Proteus®ONE rotates from 325° to 188°, covering a total range of 223°, with proton beams delivered through a dedicated scanning beam nozzle. The treatment couch rotates from 0° to 180°. Beam position is controlled by two scanning magnets, operating in the *x*-direction (left-to-right) and *y*-direction (superior-to-inferior). During commissioning, the focal lengths of the proton beams were measured to be 294.5 cm and 910.7 cm for the *x*- and *y*-directions, respectively. The clinical beam energy of the IBA Proteus®ONE unit ranges from 70 MeV to 226 MeV, with an in-air spot size (*σ*) varying from 3.44 mm at 226 MeV to 7.71 mm at 70 MeV. An accessory drawer at the end of the beam nozzle accommodates beam-modifying accessories, such as range shifters or a snout for holding proton collimating (Lin *et al* 2024). The accessory drawer could be extended or retracted along the beam’s direction, with a maximum extension of 45.4 cm and a minimum of 17.0 cm from the gantry isocenter. For our clinic, a 4 cm water-equivalent thickness (WET) range shifter is used for treating superficial targets, further degrading the proton beam from the nozzle. The resultant spot sizes are influenced by the air gap between the range shifter and the treatment surface.

Dose calculations were performed using the RayStation v2023B Monte Carlo dose calculation engine, with an uncertainty of 0.2% and a dose grid resolution of 0.1 cm isotropic spatial resolution. The spots were optimized over 100 iterations with a spot filtering setting applied, followed by 75 additional iterations before spot filtering. A minimum spot meterset constraint of 0.012 MU was enforced, in accordance with the settings of our proton machine. Robustness was not included as an optimization objective for these plans. To ensure deliverability, we used an energy layer spacing of 0.8 cm, as currently employed clinically, and confirmed that spot spacings were greater than 0.4 cm.

### 2.2. SFRT planning geometries (phantom and clinical patient)

pSFRT plans were initially developed on 2 virtual water phantoms of simple cubic and cylindrical geometries for pGRID and pLATTICE respectively. The locations of the pSFRT targets within these phantoms were varied to allow for the variation of treatment depths within our study.

**Figure 1.**
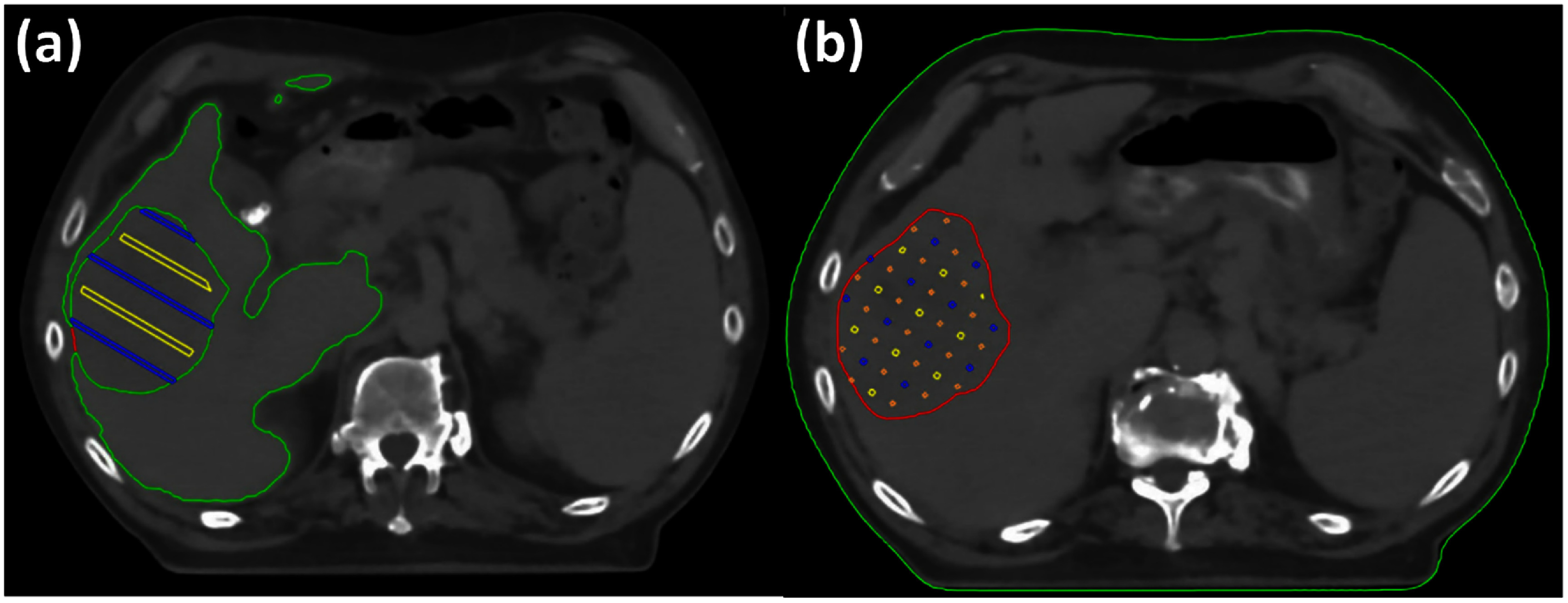
Illustration of the optimization structures used for pGRID and pLATTICE SFRT plans on a patient with locally advanced in the right hepatic lobe. Panel (a) shows the cylindrical optimization structures, and panel (b) depicts the spherical optimization structures. The color conventions for the prescription-dose structures (yellow), low dose-avoid structures (blue/cyan), and intermediate dose-avoid structures (orange) follow the same scheme as described in this figure.

In addition to the SFRT plans created for the phantom studies, we also developed clinically deliverable pGRID and pLATTICE SFRT plans retrospectively for a single patient previously treated in our Phase 1 SFRT clinical trial titled virtual GRID (vGRID) SBRT (ClinicalTrials.gov ID: NCT05727787), as depicted in figure 1, aimed at treating unresectable or metastatic hepatocellular carcinoma (HCC) with 4–12 cm tumor diameters. Patients enrolled in this trial will receive a single fraction SFRT dose ranging from 27–47 Gy using photon LATTICE techniques with the primary objective to determine the maximum tolerated dose that can be safely administered in a single fraction. The patient within this study is a 69-year-old male with locally advanced HCC in the right hepatic lobe, accompanied by extensive vascular invasion. The gross tumor volume (GTV), delineated on T2 MRI, measured 574.8 cc with the largest axial extent being 12.8 cm in the superior-to-inferior direction. The surrounding healthy liver volume was 2022.0 cc. The GTV’s closest extent to the patient’s skin was 2.7 cm in the right anterior oblique direction.

vGRID photon SFRT was generated using 6 MV flattening filter-free (FFF) beams and incorporated six partial 180° volumetric modulated arc therapy (VMAT) arcs, consisting of two coplanar and four non-coplanar arcs. The resultant photon plans had undesirable dose bridging within the GTV along with a significant integral dose spillage to the surrounding healthy liver. The main purpose of this work is to (1) develop pSFRT plans that can better confer intra-tumoral sparing by means of maximizing the intratumoral PVDR as well as volumetric integral dose sparing of the healthy liver by (2) comprehensively understanding the influences of various planning techniques (treatment depth, sphere arrangements, gantry angles, number of beams, c-t-c, sphere/cylinder diameters) on key SFRT metrics using a generalized phantom geometry and a clinical patient.

### 2.3. pSFRT planning structures and techniques

Intermediate optimization structures were created to for pSFRT treatment planning optimization within RayStation. RayStation scripting (CPython 3.8 64-bit Python Interpreter) was used to place these structures precisely and accurately. For pGRID plans, they were namely divergently-matched cylinders with 0.4–1.4 cm diameters (*D*_cyl_) and 1.7–4.3 cm center-to-center distances (c-t-c) ranging from. For pLATTICE plans, they were hexagonally-arranged spherical contours (Duriseti *et al* 2021, Kavanaugh *et al* 2022) as well as other alternative sphere arrangements incorporating cubic arrangements. All prescription-dose structures (cylinders and spheres) were confined within a 0.5 cm retraction of the GTV for safety purposes.

Optimization constraints included: (i) achieving prescription coverage of 27 Gy (RBE) to at least 99% of the prescription-dose structures within the GTV, (ii) limiting the global maximum dose to no more than 32 Gy (RBE), and (iii) preventing prescription dose spillage outside the GTV. For other defined structures, such as low-dose, intermediate-dose, void-dose avoidance, skin, healthy liver, and normal tissues, we aimed to minimize doses to the as-low-as-reasonably-achievable (ALARA) level.

In addition to the 2-, 3-, and 4-field configurations used for pLATTICE SFRT plans, we conducted a preliminary investigation into the potential benefits of using discrete arcs to enhance SFRT planning metrics. The proton arc length that was chosen had a span that included the static gantry angles that were used.

### 2.4. Design of digital brass aperture collimator

**Figure 2.**
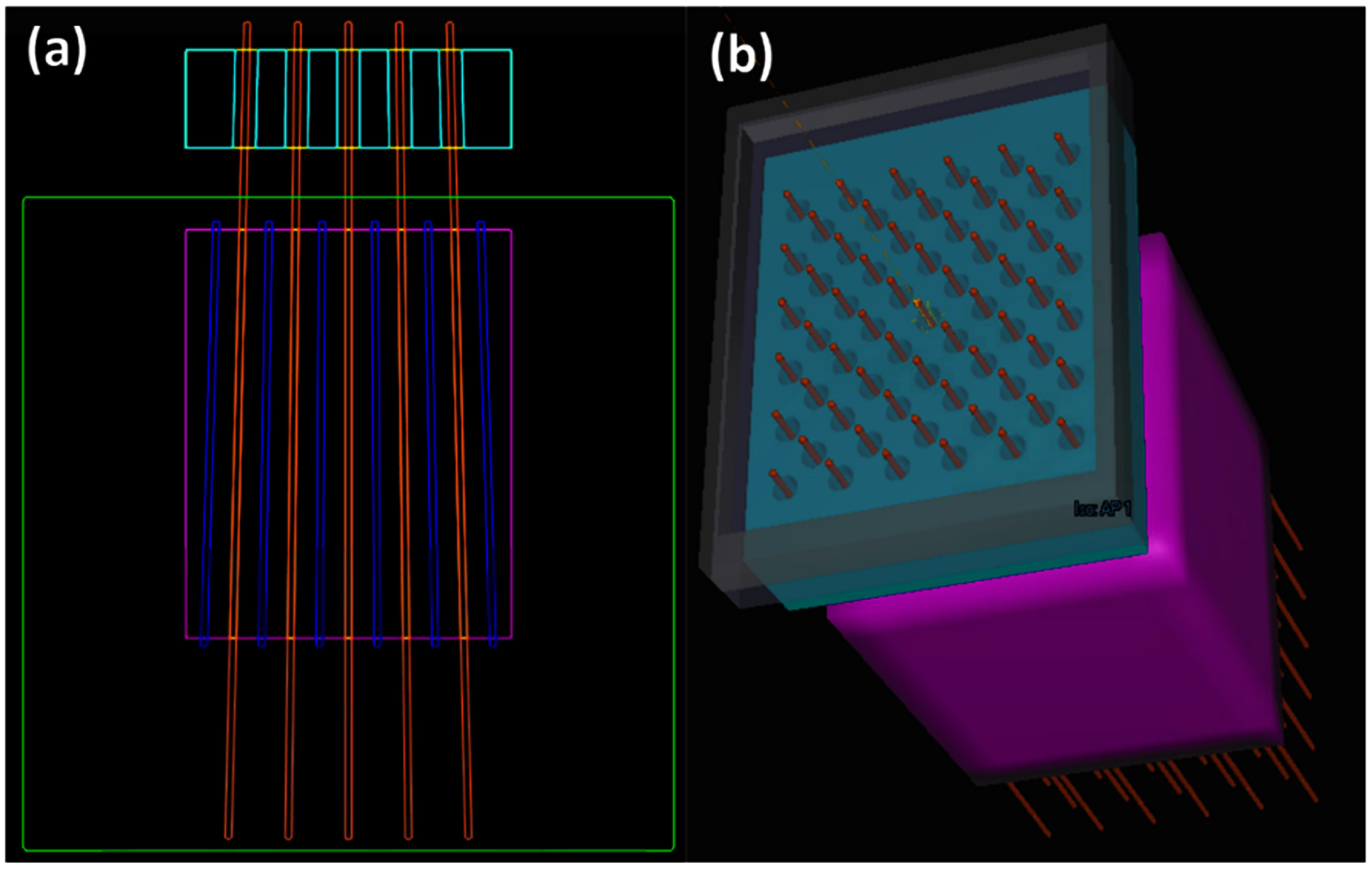
Visualization of collimating brass aperture structures (cyan contour) utilized for pGRID SFRT plans. These apertures were formed by extending prescription-dose cylinder structures upstream and cropping them from a 6 cm thick brass rectangular block, resulting in apertures that are divergently matched to the proton beam.

Preliminary investigations were conducted to assess the potential benefits of incorporating a brass collimating aperture to enhance the PVDR in pGRID plans. This evaluation was performed on the deeper pGRID phantom geometry. To fabricate the brass collimating aperture, a virtual brass support structure, 6 cm thick, was positioned directly above the gantry’s nozzle. An air gap of 7 cm was maintained to ensure adequate clearance with the phantom. Subsequently, divergently matched prescription-dose cylinders were back projected upstream towards the brass block and a 0.5 cm uniform expansion of these cylinders were subtracted from the brass block to form the divergently matched apertures, as illustrated in figure 2. pGRID plans incorporating these collimators were developed by modeling the resultant brass structures as a ‘Support Structure’ and re-optimizing with the same objectives applied to pGRID SFRT plans without an aperture.

### 2.5. SFRT plan quality assessment

Relevant SFRT metrics were evaluated for all SFRT plans according to the recommendations provided by Zhang *et al* (2020). These metrics include the generalized equivalent-uniform-dose (gEUD) of the GTV, the maximum dose to the 0.3 cm skin rind (*D*_max_), and the mean doses (*D*_mean_) to all intermediate SFRT-specific structures, including prescription-dose cylinders and spheres, as well as their corresponding low-, intermediate-, and void-dose avoidance structures. For the clinical SFRT planning, we also reported the *V*_5Gy_ of the healthy liver structure and the normal-tissue-complication-probability (NTCP). The PVDR was calculated as the ratio of the mean dose (*D*_mean_) of the prescription-dose structures to the mean dose of the avoidance structures in the SFRT plans.

**Figure 3.**
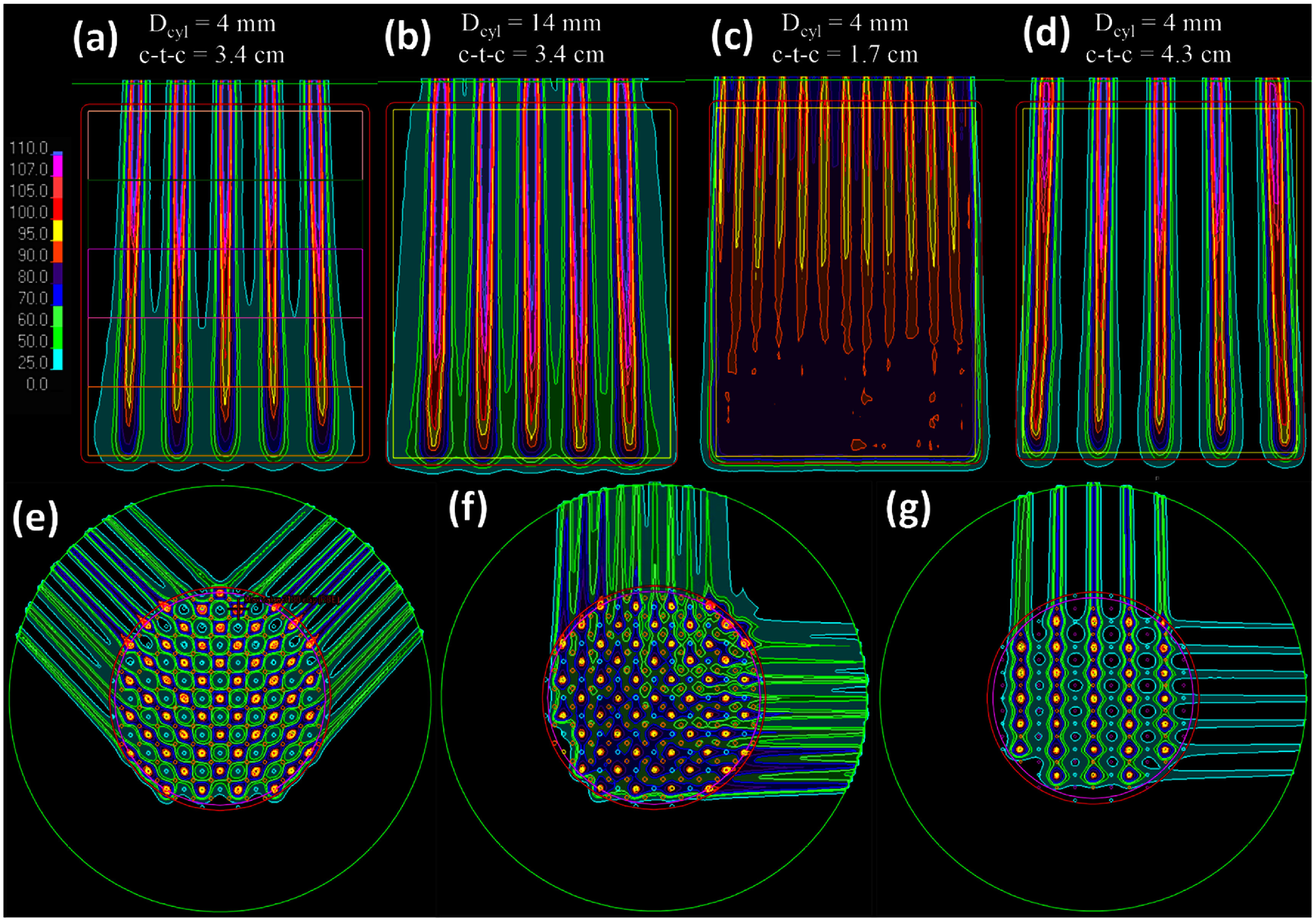
Axial dose distributions for various phantom SFRT plans namely (a)–(d) pGRID plans with different cylinder diameters (*D*_cyl_) and center-to-center (c-t-c) distances and (e), (f) pLATTICE plans with different beam orientations and sphere arrangements. For the pGRID plans, the GTV was subdivided into five bands of equal thickness along the beam’s direction for assessing SFRT metrics by depth, as shown in (a). pLATTICE plan quality was found to be highly dependent on the beam orientation; (e) illustrates the use of favorable beam angles where the dose-avoid structures are aligned parallel to the beam entrances and (f) illustrates the use of the worst possible beam angles. (g) illustrates the creation of void doses for enhanced PVDR with a mixed hexagonal/cubic sphere arrangement.

For the pGRID SFRT plans created for the phantom geometries, we assessed the depth dependence of the SFRT metrics. This involved dividing the GTV along the beam’s direction (gantry angle 0°) into five equivalent bands, as illustrated in figure 3(a), and scoring the relevant SFRT metrics within each band. Additionally, we re-optimized the pGRID plans to target only a sub-volume of the prescription dose cylinders confined within each scoring band, while maintaining intra-tumoral doses at ALARA. These metrics were then compared to those obtained from pGRID plans optimized to cover the entire length of the cylinder.

The GTV gEUD was calculated using the modified linear quadratic (MLQ) model (Niemierko 1997, Guerrero and Li 2004) with the following values for primary HCC tumors: (i) $\alpha $ = 0.010 ± 0.001 Gy^−1^, (ii) $\alpha /\beta $ = 15.0 ± 2.0 Gy and (iii) $T$ = 128 d (Tai *et al* 2008). The NTCP of the healthy liver was calculated using the Lyman-Kutcher-Berman (LKB) model with the following values for healthy liver: (i) $a$ = 0.09, (ii) $m$ = 0.7 and (iii) D_50_ = 7.5 Gy (Pursley *et al* 2020).

All pSFRT plans retrospectively developed for the clinical trial patient were compared with the corresponding photon vGRID SFRT plan that was clinically administered. While we clinically desired pSFRT plans with maximal PVDR values and healthy liver sparing, we imposed an additional dose objective whereby the pSFRT plans had to deliver similar amounts of radiation doses to the GTV. As such, the gEUD as previously defined, was used as the basis for comparing the magnitudes of SFRT doses delivered between photons (vGRID) and pSFRT. This approach prevents us from artificially creating pSFRT plans that were too sparse which will naively bias our comparisons in favor of proton modalities.

## 3. Results

### 3.1. Proton GRID SFRT metrics (phantom)

Figures 3(a)–(d) illustrate the axial dose distributions for the pGRID phantom across various c-t-c distances and cylinder diameters showing improvements in PVDR values with decreasing *D*_cyl_ and increasing c-t-c. Figures 3(b) and (c) show extremes of inadequate spatial fractionation with large *D*_cyl_ and c-t-c respectively. Figure 3(d) shows high PVDR values at 0.4 cm *D*_cyl_ and 4.3 cm c-t-c but with a sparse dose distribution resulting in reduced GTV *V*_27Gy_ and EUD. Figure 3(a) depicts a favorable trade-off (*D*_cyl_ = 0.4 cm, c-t-c = 3.4 cm) providing a reasonable PVDR with adequate EUD coverage to the GTV.

**Figure 4.**
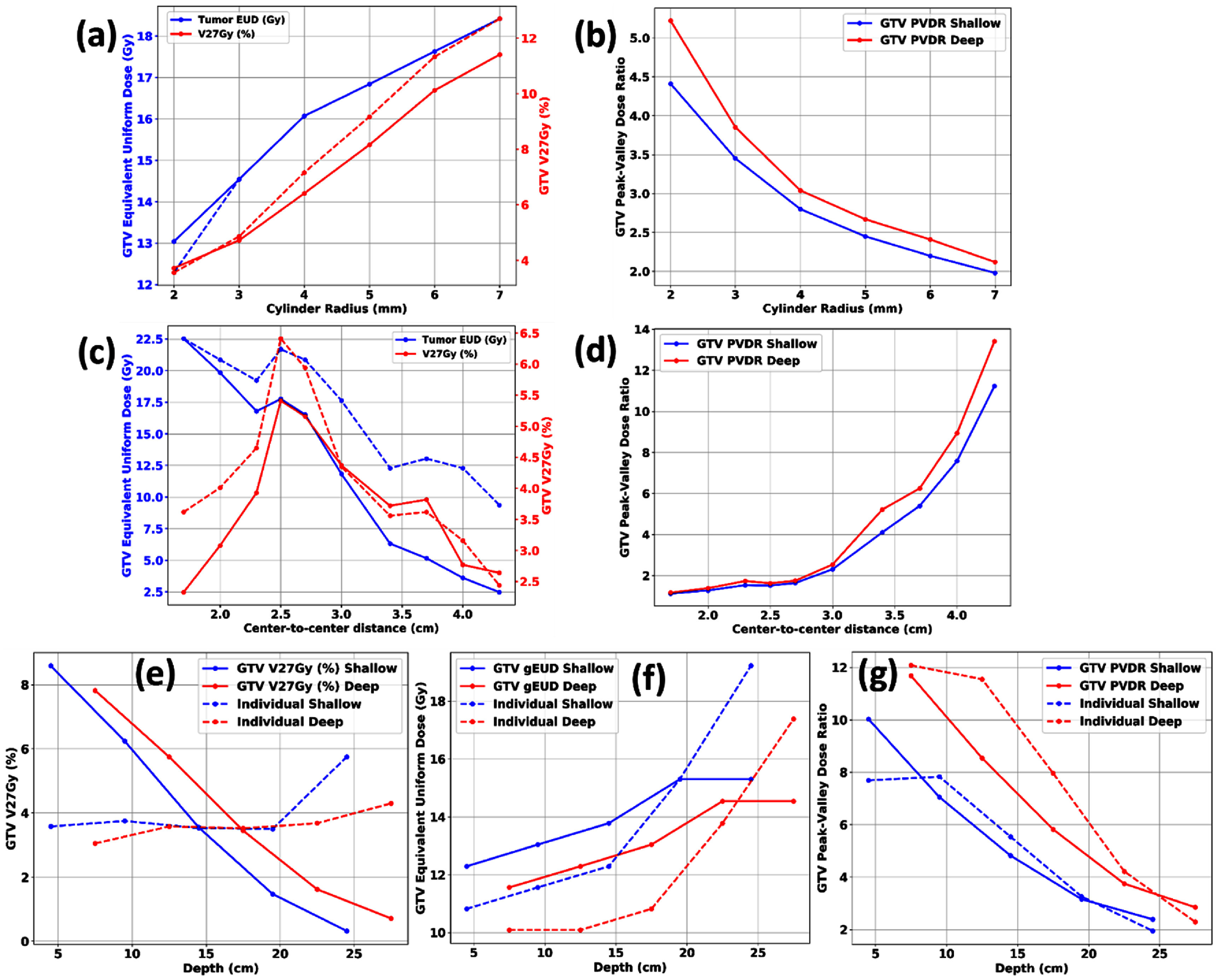
Graphs illustrating the dependency of SFRT and dose metrics, including GTV EUD, *V*_27Gy_, and PVDR, on *D*_cyl_, c-t-c and treatment depths. In (a) and (c), solid lines represent shallow pGRID targets, while dashed lines denote deeper pGRID targets. For (e) and (f), solid lines represent SFRT coverage optimization applied to the entire length of the prescription-dose cylinder while dashed lines correspond to optimization confined to the prescription-dose cylinder sub-volumes within each band.

Figure 4 shows plots of key SFRT and dose metrics (*V*_27Gy_, GTV gEUD, and PVDR), as functions of *D*_cyl_ and c-t-c describing the relationships that were mentioned previously. PVDR values were higher when the range shifter was not used which showed that small spot sizes were correlated with higher PVDR values. A correlation between EUD and *V*_27Gy_ was generally observed. However, this correlation was lost with significant intermediate dose bridging under small c-t-c values. From these plots, a minimum c-t-c threshold of 2.5 cm was identified beyond which there would be a notable decline in SFRT plan quality due to bridging.

PVDR values were generally shown to decrease with increasing depth of treatment (figures 4(e)–(g)) due significant intermediate dose bridging at deeper depths. This bridging constrained the effective range for pGRID treatments depending on the PVDR values to be delivered. In addition, the PVDR values are approximately similar between the original and re-optimized pGRID plans (figure 4(g)), suggesting that the PVDR is not influenced by the length pGRID cylinders.

### 3.2. Proton LATTICE SFRT metrics (phantom)

Figures 3(e)–(g) showed the axial dose distributions of various pLATTICE planning techniques. If a pair of non-optimal gantry angles were chosen, there will be significant dose bridging (figure 3(f)) as compared to optimal gantry angles (figure 3(e)). It was also determined that the number of gantry angles did not influence the pLATTICE plan quality if the set included the optimal angles. The pLATTICE PVDRs can be increased by using non-optimal sphere packing patterns such as cubic patterns (figure 3(g)). As seen, there are recurrent dose voids with these cubic structures.

### 3.3. Proton GRID SFRT metrics (liver patient)

**Figure 5.**
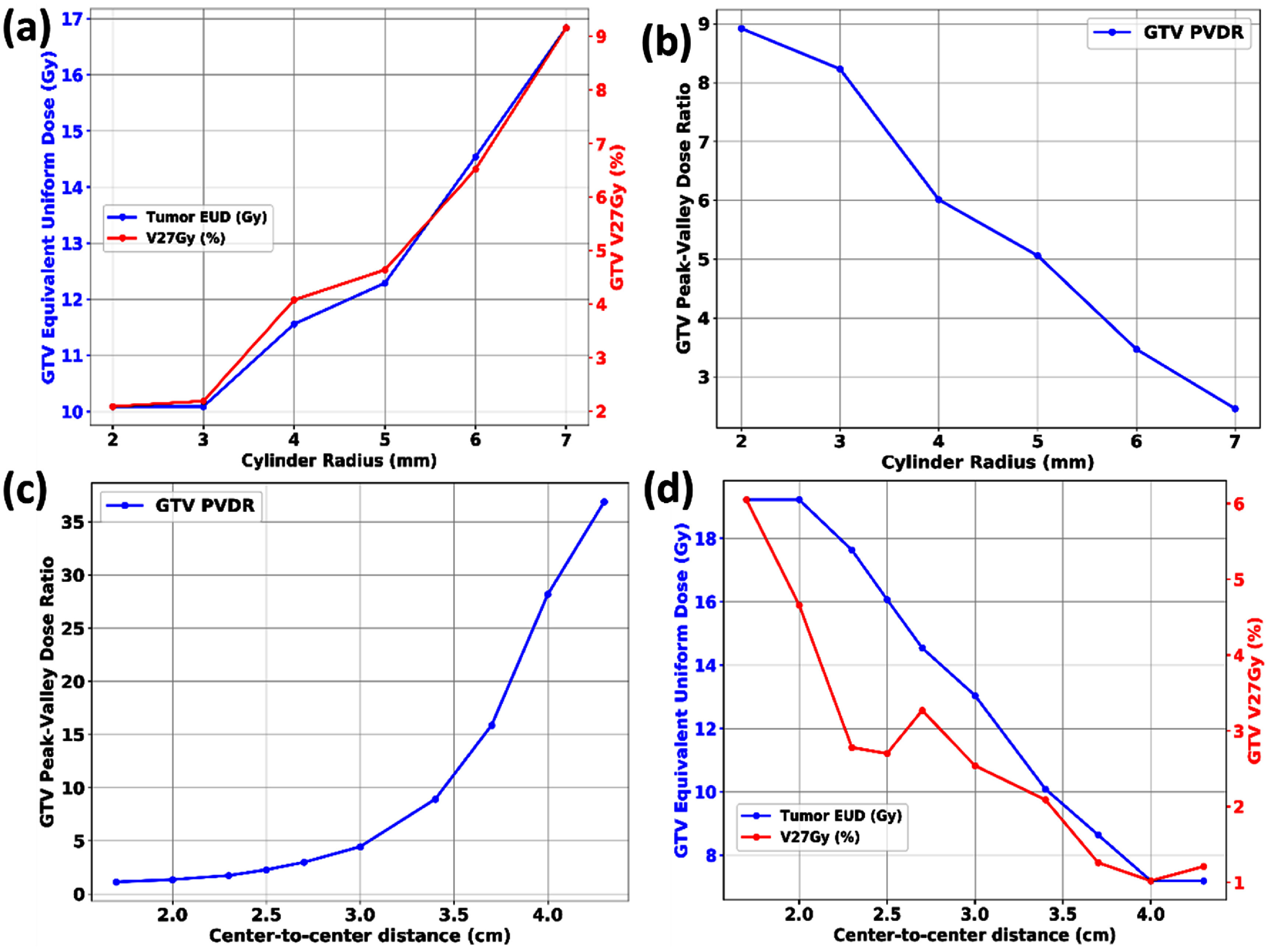
Plots showing the dependence of SFRT and dose metrics, including GTV EUD, *V*_27Gy_, and PVDR, on cylinder radius and c-t-c distances for the clinical HCC patient. Loss of correlation between *V*_27Gy_ and GTV EUD is observed with significant intermediate dose bridging.

**Figure 6.**
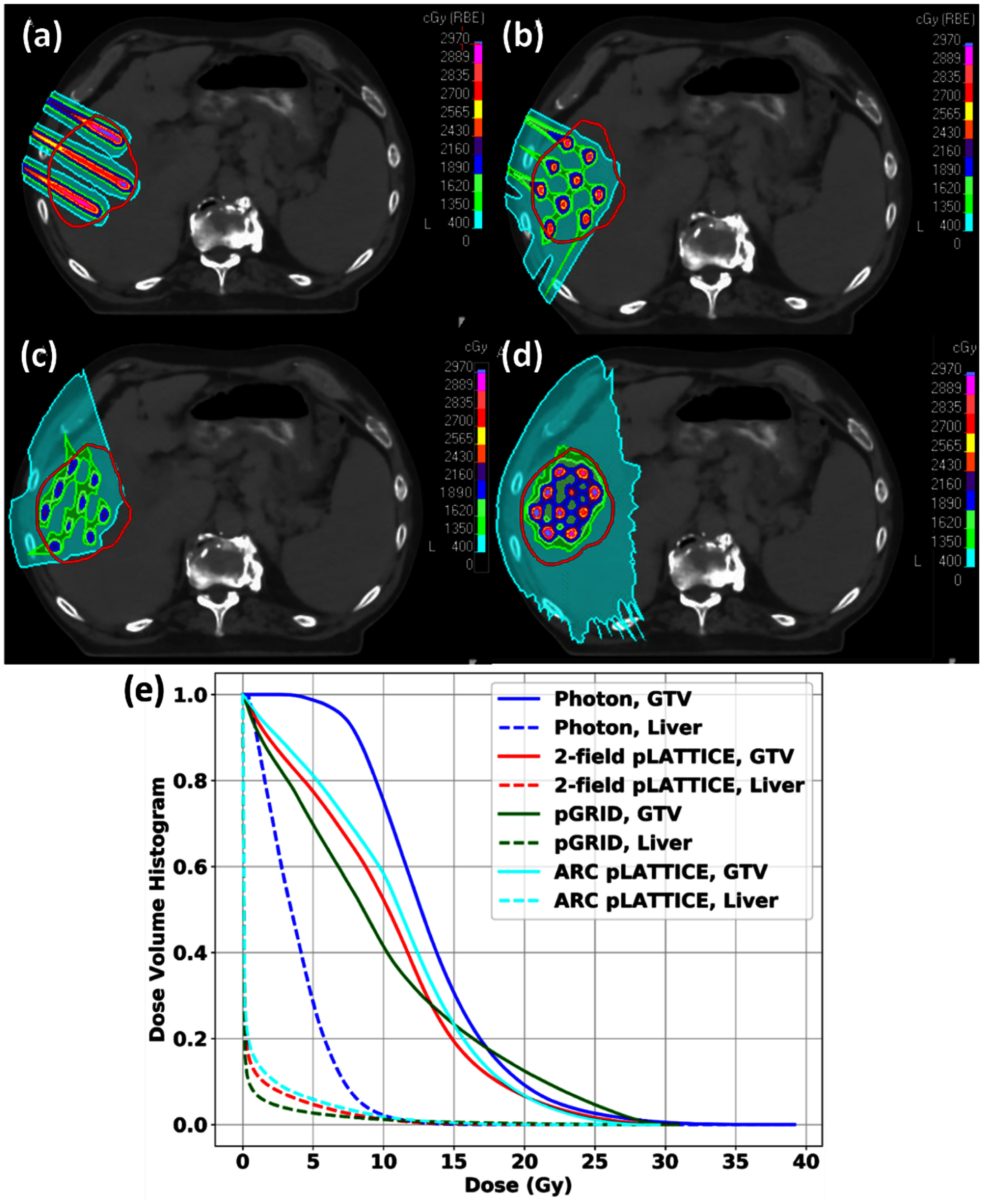
Illustrations of the axial dose distributions and dose-volume histogram (DVH) plots for various clinical SFRT planning techniques. (a): pGRID, (b), (c): pLATTICE plans with a pair of (b): favorable and, (c): non-favorable beam angles. (d): vGRID photon SFRT.

Figure 5 shows the dependencies of key pGRID SFRT metrics (*V*_27Gy_, gEUD, PVDR) with varying *D*_cyl_ and c-t-c values for the clinical patient. Similar phenomena were noticed, namely the *V*_27Gy_ and gEUD correlation, breakage of correlations with significant dose bridging. Notably, the *V*_27Gy_ for the vGRID photon SFRT plan is 1.50% as compared to 2.09% for the pGRID plan (3.4 cm c-t-c, 0.4 cm *D*_cyl_). pGRID PVDR is 8.92, representing a three-fold improvement compared to the corresponding vGRID photon PVDR values which are approximately 2.7–3.0. The axial dose distribution of the pGRID patient plan is shown in figure 6(a).

### 3.4. Proton LATTICE SFRT metrics (liver patient)

Figures 6(b) and (c) shows the resultant axial dose distributions for pLATTICE SFRT plans for the clinical HCC patient demonstrating the concept of the favorable beam angles (figure 6(b)) and the resultant intermediate dose bridging that arises with the choice of unfavorable angles (figure 6(c)).

Table 1 contains the key SFRT and dose metrics for various pSFRT techniques for the clinical patient. pLATTICE planning techniques resulted in a lower skin D_0.3cc_ with multiple beam angles. PVDR decreased with an increasing number of fields and if suboptimal beam angles were used. Cubic arrangements of spheres increased the GTV PVDR, albeit with a moderate decrease of the GTV *V*_27Gy_.

**Table 1.** Dosimetric parameters for proton LATTICE (pLATTICE) plans (27 Gy prescription) for a hepatocellular carcinoma patient as a function of the number of gantry angles, using a fully hexagonal sphere arrangement. Parameters *D*_mean,high-dose_, *D*_mean,interm-dose_, and *D*_mean,low-dose_ represent the mean doses from high-, intermediate-, and low-dose optimization pLATTICE spheres, respectively, as shown in figure 1. This table also includes dose metrics for photon LATTICE and proton GRID (pGRID) SFRT plans for comparison, and reports on two additional sphere arrangements, highlighting the potential for co-optimization of sphere sizes, packing densities, patterns, and placements.

	Comparison plans		pLATTICE Plans						
			Full Hexagonal						
	Photon	pGRID	2-field	3-field	4-field	2-field unfavorable	pLATTICE ARC	2-field Mixed Cubic	2-field Full Cubic
Tumor EUD	13.04 Gy	10.09 Gy	10.09 Gy	10.09 Gy	10.09 Gy	11.56 Gy	10.82 Gy	7.91 Gy	7.19 Gy
Liver gEUD	3.17 Gy	0.22 Gy	0.27 Gy	0.28 Gy	0.29 Gy	0.27 Gy	0.29 Gy	0.24 Gy	0.22 Gy
*D*_mean,high-dose_	N/A	28.18 Gy	28.33 Gy	28.36 Gy	28.32 Gy	25.07 Gy	26.60 Gy	27.83 Gy	27.30 Gy
*D*_mean,interm-dose_	N/A	N/A	9.56 Gy	9.66 Gy	9.59 Gy	10.48 Gy	10.19 Gy	8.91 Gy	9.17 Gy
*D*_mean,low-dose_	N/A	3.16 Gy	6.80 Gy	6.94 Gy	7.11 Gy	10.86 Gy	8.47 Gy	5.54 Gy	4.11 Gy
*D*_mean,void_	N/A	N/A	N/A	N/A	N/A	N/A	N/A	3.21 Gy	2.26 Gy
PVDR_interm_	N/A	N/A	2.96	2.94	2.95	2.39	2.61	3.12	2.98
PVDR_low_	2.7-3.0	8.92	4.17	4.09	3.98	2.31	3.14	5.02	6.64
PVDR_void_	N/A	N/A	N/A	N/A	N/A	N/A	N/A	8.67	12.08
*V*_5Gy,Healthy liver_	574.62 cc	53.55 cc	94.18 cc	94.34 cc	96.22 cc	106.63 cc	118.46 cc	65.78 cc	47.45 cc
Skin D_0.3cc_	7.79 Gy	23.24 Gy	14.68 Gy	14.20 Gy	13.13 Gy	13.90 Gy	11.94 Gy	13.87 Gy	14.89 Gy
MU	N/A	5801.79	6232.65	6393.06	6549.41	7385.11	7395.37	4663.80	4357.97
#Spheres	40	15^a^	36	36	36	36	36	26	27

^a^ Refers to number of cylinders within the GTV (0.4 cm diameter, 3.4 cm c-t-c distances).

Figure 6(e) presents the dose-volume histograms (DVHs) for the GTV and healthy liver across all pSFRT techniques investigated. Photons resulted in the highest integral dose to the healthy liver while all pSFRT methods showed superior intratumoral sparing with equivalent gEUD deliveries to the GTV.

### 3.5. Dosimetric effects of brass aperture collimator (phantom)

**Figure 7.**
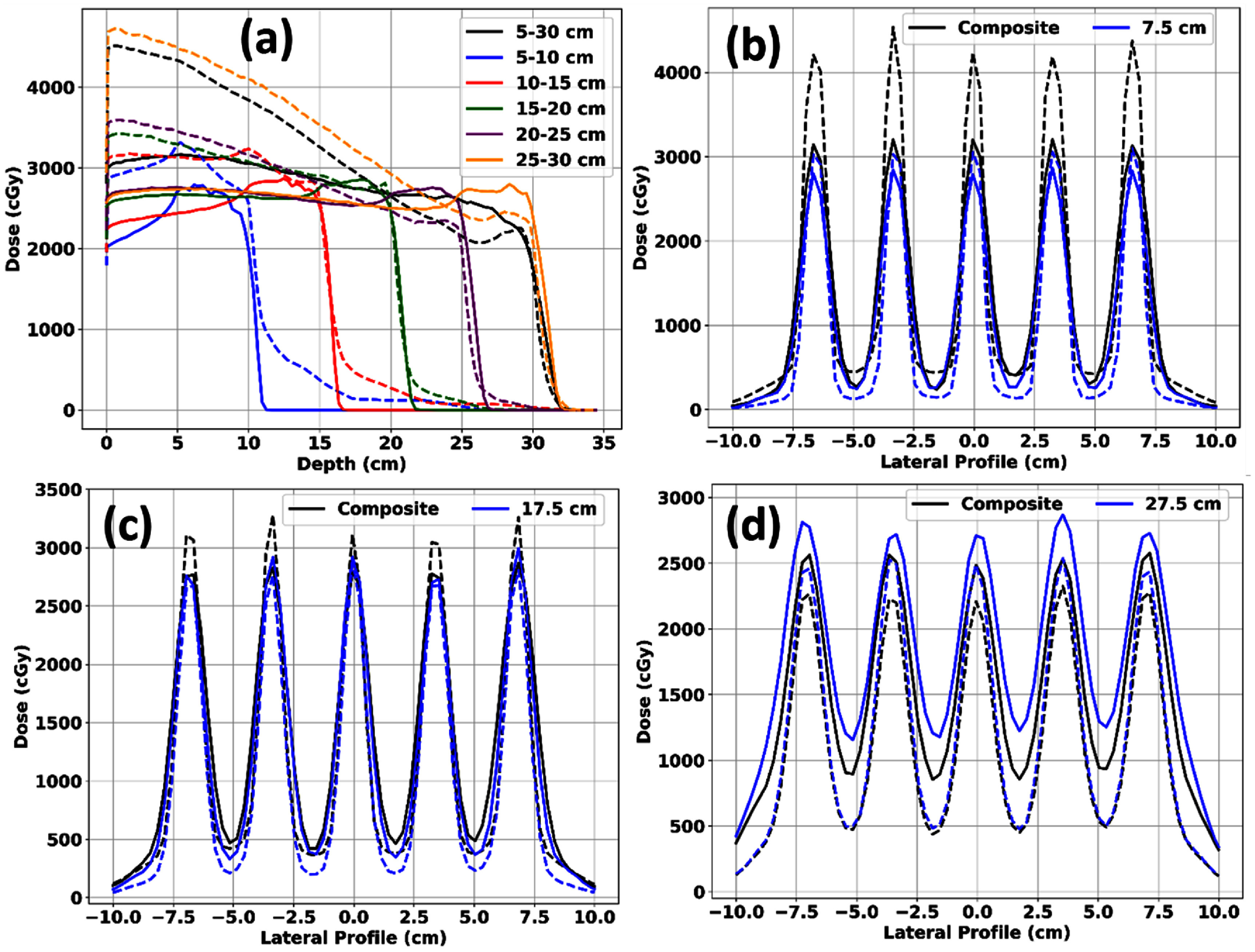
(a) Percentage depth-dose (PDD) curves and (b)–(d) lateral profiles of pGRID plans with and without a brass aperture. Solid lines represent pGRID plans without an aperture, while dashed lines indicate plans with a brass aperture. Black lines show PDDs and profiles at various depths for pGRID plans with a 27 Gy coverage objective over the entire length of the cylinder, whereas colored lines correspond to plans with coverage objectives confined to the subvolume of the cylinder.

Finally, we present data for pGRID plans utilizing a brass collimator aperture, specifically for the deep pGRID phantom. Figure 7(a) displays the percentage depth dose (PDD) profiles for the pGRID plans with the brass collimator. Figures 7(b)–(d) illustrate the lateral dose profiles through the beam’s isocenter at depths of 7.5 cm, 17.5 cm, and 27.5 cm, respectively. The use of the brass collimator resulted in an increased skin *D*_0.3cc_, reaching up to 54.66 Gy (RBE) for deeper treatments, as shown in figure 7(a). Nevertheless, the brass collimator also enhanced the PVDR at deeper depths, achieving a PVDR value of 4.85 at 27.5 cm depth, as depicted in figure 7(d).

## 4. Discussion

### 4.1. Practical pSFRT clinical planning techniques

In our study, we did not investigate *D*_cyl_ that were smaller than 0.4 cm; their use resulted in partial volume artifacts manifesting as disjointed cylindrical contours which resulted in poorer pSFRT planning qualities. These issues can be mitigated with CT datasets with smaller slice thicknesses or minimum *D*_cyl_ threshold. In our study, the digital phantom and patient CT datasets had slice thicknesses of 0.1 cm and 0.2 cm respectively and 0.4 cm *D*_cyl_ is constrained by the latter slice thickness. We recommend utilizing 0.1 cm slice thicknesses for clinical pSFRT treatment planning and conducting a thorough evaluation of the generated cylinder and sphere contours during the planning process.

Optimal beam entries aligned the pLATTICE dose-avoid structures parallel to the beam’s direction which allowed for optimal dose sparing and superior PVDRs. In contrast, sub-optimal beam angles forced the optimizer to deliver proton doses through these dose-avoid structures. For clinical practice, we recommend conducting an initial assessment of the beam directions for planning purposes. Proximal OARs relative to the GTV or physical clearance with the couch and patient immobilization devices will affect the available angles. If required, non-coplanar beam entries may also be considered as well. Once the beam directions have been selected, the pLATTICE SFRT contours can be generated and a quick assessment of the projection alignment of the dose-avoid structures can be assessed using the beam’s eye view of the TPS. Additionally, our results showed that an increased number of pLATTICE beam angles and discrete arcs did not noticeably influence the SFRT plan qualities. We recommend using the two-field pLATTICE method to improve the deliverability of the pLATTICE plans.

Deeper treatment depths led to PVDR decreases due to dose bridging caused by increased spot sizes with increasing depth. This effect was also similarly seen if a range shifter was used. Practically, there is a maximum pGRID treatment depth of approximately 25 cm WET beyond which the PVDR falls below 3. At deep depths, we recommend alternative approaches such as pLATTICE or adjustments in *D*_cyl_ and c-t-c to achieve the desired PVDR. Additionally, it will be challenging to treat shallow and deep targets concurrently due to the lateral broadening effect caused by the range shifter, which reduces the achievable PVDR across the GTV. Investigations into the mixed usage of range shifters for such treatments will be performed in future studies.

### 4.2. Effects of number of different sphere arrangements on pLATTICE treatments

Alternative sphere arrangements incorporating cubic structures achieved lower sphere packing densities by approximately 28%. Similar to fully hexagonal arrangements, a pair of optimal pLATTICE beam angles can always be found. Our results demonstrate an increase in the PVDR from 4.17 to 6.64 with a decrease in the *V*_27Gy_ from 0.61% to 0.21% for the clinical patient planned with fully-cubic pLATTICE arrangements. Future works will involve recovering clinically desired GTV *V*_27Gy_ and EUD metrics by a further fine-tuning of the spherical diameters and c-t-c for these alternative sphere arrangements.

### 4.3. Comparisons between SFRT techniques (pGRID, pLATTICE, vGRID)

Our results show all pSFRT techniques demonstrating a significant reduction in the healthy liver *V*_5Gy_ from 574.62 cc (vGRID) to 53.55 cc (pGRID) and 94.18 cc (2-field pLATTICE). Alternative pLATTICE arrangements can attain lowered *V*_5Gy_ of 65.78 cc (mixed hexagonal/cubic) and 47.45 cc (fully cubic) due to their lower sphere packing densities. These results highlight the superior capability of pSFRT in reducing integral doses to adjacent OARs which is clinically beneficial for meeting volumetric dose objectives for parallel-type OARs such as the liver and for re-irradiation scenarios.

Similar investigations have been performed recently; Mossahebi *et al* demonstrated a proton LATTICE planning approach using two cardinal orthogonal beams to create vertices with a 1 cm proton vertex diameter and spaced 3–3.5 cm between each vertex (2024). Compared with photon approaches, proton LATTICE was demonstrated to have higher PVDRs, spatial dose heterogeneities and modulations and achieved a greater sphere packing. Grams *et al* compared uncollimated and collimated proton GRID approaches with photon SFRT treatments and came to the same conclusion where proton GRID plans attained the highest dose heterogeneities as compared with photon plans. However, they concluded that photon plans achieved higher EUD values as compared to their proton counterparts (2022).

### 4.4. Effects of brass apertures on pGRID treatments

While brass apertures can enhance the PVDR at both shallow and deep treatment depths due to improved collimation (figure 7), their clinical application is complicated by the relatively high surface doses associated with pGRID plans (figure 7(a)). This surface dose enhancement becomes especially pronounced at depths greater than 15 cm, regardless of the cylindrical sub-volume length being treated. Therefore, if brass apertures are utilized, it is advisable to carefully assess the maximum doses to the skin and potentially adjust treatment prescriptions or consider alternative SFRT techniques to mitigate these high surface doses.

### 4.5. Challenges and limitations

In this study, the dosimetric impact of robustness optimization was not investigated. If coverage objectives were optimized robustly to account for setup and range uncertainties, the resultant PVDR would likely decrease due to the need for over-coverage of the defined contours. This effect would be further accentuated with the use of multiple gantry angles, potentially skewing outcomes in favor of pGRID or two-field pLATTICE techniques. Our current study design focuses purely on geometrical considerations, without accounting for these robustness factors. However, preliminary robustness optimizations were performed at 3.5% range uncertainties for two-field pLATTICE plans. The resultant PVDRs for the low- and intermediate-dose spheres were found to decrease to 3.08 and 2.31 respectively due to the dose smearing effects. Future works will be dedicated to (1) better understanding the sensitivities and resultant influences of the PVDR with plan robustness and (2) determining an appropriate robustness criteria and/or even pSFRT contour generation strategies to be applied during treatment planning to ensure that the desired PVDRs are created with the pSFRT plan delivery.

While clinical robustness is less of a concern for pGRID, it is clinically essential to minimize pLATTICE dose smearing which can degrade the PVDR (Ginn *et al* 2024). While spirometers (Pakela *et al* 2022, Sabouri *et al* 2024) can be used to mitigate these effects, a potential solution is to deliver the full prescription dose to each high-dose sphere using a single beam angle, rather across multiple angles. Developing novel optimization algorithms to support this approach will be a subject of future research. OAR robustness is indirectly included in this study by limiting high-dose sphere and cylinder placements 0.5 cm from the GTV’s surface.

Additionally, our study did not address the potential effects of linear energy transfer (LET) differences between pGRID and multi-field pLATTICE plans, or the presence of high LET regions within distal OARs. These effects, along with potential LET optimization strategies, will be explored in future investigations.

The clinical usability of these pSFRT techniques is contingent on the dose calculation accuracies of the RayStation TPS especially at small fields. This will be addressed in future works which will investigate (1) the suitability of various detectors for small field proton dosimetry along with their associated limitations, (2) a comprehensive experimental commissioning of the pSFRT techniques and (3) the development of an expedient patient-specific quality assurance (QA) protocol for routine use. Preliminary investigations into the accuracy of the RayStation TPS’ proton small field dosimetry accuracy was performed with experimental measurements using cross-calibrated small field detectors which showed dose agreements within ±2% for field sizes that are as small as 0.5 × 0.5 cm^2^ consisting of 4 proton spots.

While we have used a patient for our study, the broad pSFRT treatment planning lessons that are learned such as the pre-selection of 2 ideal and orthogonal beam angles, the generation of SFRT structures with respect to the initial beam angles, the choice of smallest possible diameter and tailoring the c-t-c values to the desired tumor gEUD will be generally applicable to a wide range of anatomies. Future works will involve the creation of pSFRT plans using these approaches and principles on a wide range of anatomies and assessing the suitability of pSFRT methods to treat other diseases.

### 4.6. Future studies

Future work will focus on experimental measurements to confirm their clinical deliverability. Novel proton arc optimization algorithms to potentially enhance the quality of pLATTICE SFRT plans will be investigated. Alternative pLATTICE sphere arrangements along with the fine-tuning of sphere diameters and c-t-c to achieve clinically desirable SFRT and dose metrics will be investigated. The impact of LET distributions within the GTV with different SFRT planning methodologies will be investigated along with different RBE calculation models. Finally, the effects of range and intra-fractional motion uncertainties on multi-field pLATTICE plans along with the efficacies of potential strategies to mitigate these issues will be evaluated.

## 5. Conclusion

In this study, we developed various pSFRT treatment planning methods to enable clinical SFRT treatments on an IBA Proteus®ONE synchrocyclotron. We demonstrated the ability of pSFRT to achieve comparable GTV V_27Gy_ and EUD values with vGRID plans with up to a three-fold improvement in the PVDR. For pLATTICE treatment planning, we demonstrated the critical role of sphere orientations relative to beam entrances. All pSFRT approaches provided superior healthy liver sparing compared to vGRID. Preliminary studies of alternative non-hexagonal sphere arrangements suggest potential improvements to achievable pLATTICE PVDR and will be investigated in future works.

## Data Availability

All data that support the findings of this study are included within the article (and any supplementary information files).
